# Design and Construction of Enzyme-Based Electrochemical Gas Sensors

**DOI:** 10.3390/molecules29010005

**Published:** 2023-12-19

**Authors:** Wenjian Zhang, Xinyi Chen, Yingying Xing, Jingqiu Chen, Lanpeng Guo, Qing Huang, Huayao Li, Huan Liu

**Affiliations:** 1 School of Integrated Circuits, Wuhan National Laboratory for Optoelectronics, Optics Valley Laboratory, Huazhong University of Science and Technology, 1037 Luoyu Road, Wuhan 430074, China; zhangwenjian@hust.edu.cn (W.Z.); chenxinyi0810@outlook.com (X.C.); m202371380@hust.edu.cn (Y.X.); zian_chen@hust.edu.cn (J.C.); guolanpeng@163.com (L.G.); huangqingcl@163.com (Q.H.); huayaoli@hust.edu.cn (H.L.); 2 Wenzhou Key Laboratory of Optoelectronic Materials and Devices Application, Wenzhou Advanced Manufacturing Institute of HUST, 1085 Meiquan Road, Wenzhou 325035, China

**Keywords:** enzyme electrode, interface, electrochemical gas sensor, specificity

## Abstract

The demand for the ubiquitous detection of gases in complex environments is driving the design of highly specific gas sensors for the development of the Internet of Things, such as indoor air quality testing, human exhaled disease detection, monitoring gas emissions, etc. The interaction between analytes and bioreceptors can described as a “lock-and-key”, in which the specific catalysis between enzymes and gas molecules provides a new paradigm for the construction of high-sensitivity and -specificity gas sensors. The electrochemical method has been widely used in gas detection and in the design and construction of enzyme-based electrochemical gas sensors, in which the specificity of an enzyme to a substrate is determined by a specific functional domain or recognition interface, which is the active site of the enzyme that can specifically catalyze the gas reaction, and the electrode–solution interface, where the chemical reaction occurs, respectively. As a result, the engineering design of the enzyme electrode interface is crucial in the process of designing and constructing enzyme-based electrochemical gas sensors. In this review, we summarize the design of enzyme-based electrochemical gas sensors. We particularly focus on the main concepts of enzyme electrodes and the selection and design of materials, as well as the immobilization of enzymes and construction methods. Furthermore, we discuss the fundamental factors that affect electron transfer at the enzyme electrode interface for electrochemical gas sensors and the challenges and opportunities related to the design and construction of these sensors.

## 1. Introduction

As the core component of artificial smell, gas sensors are applied in fields such as environmental monitoring, public health safety, medical health, food safety, and military aerospace [1]. Over the past few decades, various types of gas sensors have been developed based on different sensing materials and device structures. Accordingly, they are divided into semiconductor gas sensors, electrochemical gas sensors, catalytic combustion gas sensors, solid electrolyte gas sensors, infrared absorption gas sensors, ionized gas sensors, and resonant gas sensors. The main parameters of gas sensors, including sensitivity, response time, recovery time, and repeatability, were used for evaluation. However, their cross-sensitivity to a variety of gases hinders the qualitative identification and quantitative detection of mixed gases [2,3]. Biomolecules, with their excellent specificity, described with a “lock-and-key”, such as enzyme–substrate, antigen–antibody, and ligand–receptor mutual discrimination and specific reactions, provide a new paradigm for designing high-specific and high-sensitivity gas sensors. Nowadays, biomaterials include specific enzymes [4], insect antennae [5], odor-binding proteins [6], olfactory receptors [7], and sensitive peptides [8]. Based on the physiological properties of these biomaterials, highly sensitive and specific gas analysis is performed. Enzymes, which are highly specific catalytic proteins, can specifically catalyze reactions. In 1962, Clark innovatively combined glucose oxidase (GOD) with an oxygen electrode for the first time to construct an enzyme electrode that used enzyme molecules to identify target objects. Since then, the specific detection of target objects by electrochemical enzyme electrodes has been rapidly developed [9]. Due to the enzymes has advantages, like being simple and high-efficient, which has been widely applied for the detection of gas, such as using alcohol dehydrogenase for the determination of ethanol in beverages [10], formaldehyde dehydrogenase for monitoring formaldehyde in the air [11], and secondary alcohol dehydrogenase and a nicotinamide adenine dinucleaotide cofactor used for detecting breath acetone concentration [12].

**Figure 1:**
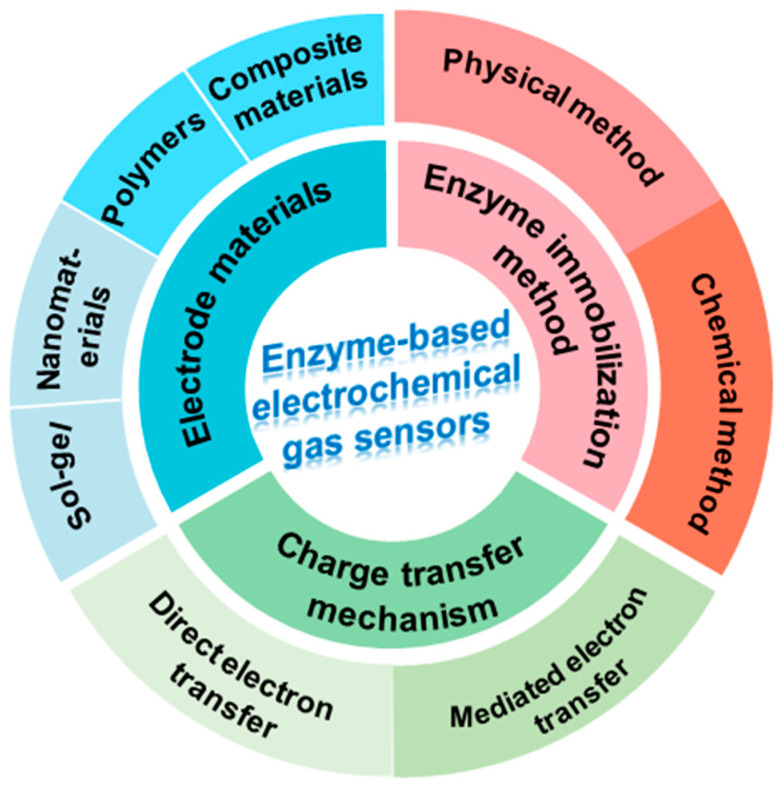
The strategies to achieve high-performance enzyme-based electrochemical gas sensors.

Electrochemical techniques are advantageous due to their simplicity and high detection efficiency. The typical electrochemical biosensor consists of an analyte, receptor, and transducer [13]. Electrochemical sensors have been widely used in gas detection by transducing the chemical reaction at the electrode–solution interface into current signals via electric potential. In the detection of gas molecules, receptors play a crucial role in enzyme-based electrochemical gas sensors. Gas molecules and enzymes are specifically compatible, leading to their binding and production of electrons as signals. Consequently, a transducer is employed to convert this signal into measurable electrical signals. The electrochemical electrode translates the chemical reaction occurring in the active component into an amperometric or potentiometric signal. Enzyme biological amperometry sensors, unlike traditional amperometry sensors, utilize enzymes as catalysts at the electrode rather than noble metals like gold, platinum, and palladium. Gas molecules are typically detected through either physical binding or a chemical reaction that occurs at the electrode [14]. However, their electroactive centers are often tightly closed or insulated by the enzyme protein body, making electron transfer between the enzyme and the electrode difficult [15]. Meanwhile, several factors influence the reaction rate and the quality of the signal transfer. It is beneficial to design electrode surfaces rationally to optimize the interaction between the enzyme and the electrode surface when considering electron transfer. The interface of enzyme electrodes in electrochemical gas sensors is particularly important for high-level development. To achieve high-performance enzyme electrochemical gas sensors, optimization strategies have been implemented, including the immobilization of the enzyme, electrode material, and charge transfer mechanism, which are graphically represented in Figure 1.

In this perspective, we summarize the recent achievements in enzyme-based electrochemical gas sensors, focusing on the design of low-cost, long-lasting, specific, and selective sensors. The methods of designing and optimizing these sensors are discussed, such as enzyme surface modification, enzyme immobilization technology, principles and methods, mediator selection, and electrode material optimization. We also highlight the development prospects of enzyme electrode amperometric gas sensors. The opportunities and challenges in practical applications of highly specific enzyme electrode sensors are also addressed. This paper aims to contribute to the design of the specificity and sensitivity of enzyme-based electrochemical gas sensors that provide a promising solution for detecting and quantifying various gases with high accuracy and reliability. Furthermore, the integration of enzyme gas sensors into IoT applications opens new possibilities for real-time, remote monitoring of air quality, industrial emissions, and workplace safety.

## 2. The Types and Principles of Enzyme Electrode Electrochemical Gas Sensor

### 2.1. Three-Electrode Enzyme Electrochemical Gas Sensor

**Figure 2:**
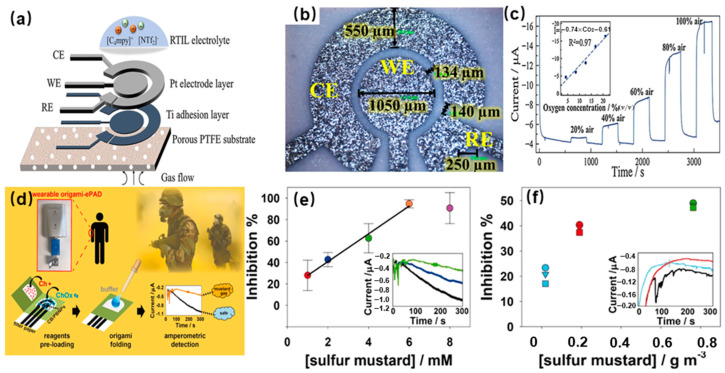
(**a**) The structure schematic of the electrochemical gas sensor; (**b**) The image of the electrodes by optical microscope; (**c**) The current response of miniaturized planar electrochemical gas sensor to oxygen [16]; (**d**) The preparation process and working principle of a wearable origami-like paper-based electrochemical biosensor for sulfur mustard detection; (**e**) The detection results of origami-ePAD for the standard solution of SM in liquid phase (absence (black) and in presence of SM concentrations equal to 2 mM and 4 mM); (**f**) The detection results of origami-ePAD for the aerosolized SM(absence (black line) and in presence of SM concentrations equal to 0.019 g/m^3^ and 0.76 g/m^3^) [21].

The three-electrode system is a classic biochemical detection technology, which has been applied for the transduction of electric signals of enzyme electrochemical gas sensors. The structure of a miniaturized planar electrochemical gas sensor is shown in Figure 2a. This system consists of a working electrode (WE) to identify the target gas molecules, a counter electrode (CE) that acts as a current source, a reference electrode (RE) that applies a stabilizing potential, and liquid electrolytes [16]. The current signal is generated by an electrochemical reaction on the WE and is used for the quantification of target gas molecules. The structure image of the electrodes by optical microscope is shown in Figure 2b. The diameter of the WE is 1050 μm, and the width of the CE and RE is 550 μm. The gap between the WE and CE is about 130 μm, and the gap between the RE and CE is 140 μm. The detection principle of the enzyme electrode electrochemical sensor for gas molecules is described thoughtfully. Firstly, the enzyme is immobilized on the surface of the WE. Secondly, gas molecule measurements are performed by immersing the three-electrode system in an electrolyte. The enzyme catalyzes the oxidation or reduction of target gas molecules, resulting in the transfer of electrons. These electrons are then transferred to the WE, leading to the production of a current that is directly related to the concentration of target gas molecules [17]. The miniaturized planar electrochemical gas sensor was applied for the detection of oxygen. The results in Figure 2c show the sensor can rapidly respond to a series of oxygen concentrations. The inset of Figure 2c shows the plot of current vs. oxygen concentration. The sensitivity of the sensor is 0.74 μA/[% oxygen], and the linearity is 0.97.

Three electrode electrochemical enzyme gas sensors are extensively utilized for gas detection, such as the formaldehyde dehydrogenase for the detection of formaldehyde [18]. The alcohol oxidase was utilized for the monitoring of ethanol [19]. The enzyme electrode of hydrogenase was designed for the detection of hydrogen [20]. An electrochemical biosensor has been applied for the detection of sulfur mustard, which is one of the most dangerous and extensively used chemical warfare agents [21]. A novel wearable electrochemical biosensor was prepared using the following steps (Figure 2d). First, the filter paper-based chemical electrodes were produced by screen-printing. Second, both choline chloride (Ch) and choline oxidase enzyme (ChOx) solutions were separately preloaded. Finally, the enzymatic reaction was activated by adding phosphate buffer solution (PBS). Sulfur mustard (SM) agent detection was carried out by monitoring their inhibitory effects toward the choline oxidase enzyme, through the amperometric measurement of the enzymatic byproduct hydrogen peroxide. The origami-like devices used to create a wearable, ready, and easy-to-use electrochemical PAD (origami-ePAD) were applied for the detection of the standard solution of SM in a liquid phase. As shown in Figure 2e, a linear between 1 and 6 mM was obtained. The inset shows the corresponding current (E  =  0.0  V and t  =  300  s) obtained from the detection of H_2_O_2_ enzymatic byproduct in the absence (black) and the presence of SM concentrations equal to 2  mM and 4  mM. This result demonstrated that the newly developed origami-ePAD is suitable for SM detection in the liquid phase. Meanwhile, the suitability of developed origami-ePAD for the detection of aerosolized SM was verified. As shown in Figure 2f, current values were sampled three times to evaluate the extent of inhibition at different exposure times, indicating the sensor can alert for the presence of airborne SM in only 60  s. This analytical approach provides a strategy applicable to the real-time monitoring of a variety of chemical weapon threats.

### 2.2. Field-Effect Transistor (FET) Enzyme Electrochemical Gas Sensor

The field effect transistor serves as a platform for chemical detection. This is built upon an electrical device, utilizing the electric field effect of the control input circuit to regulate the output circuit’s current. This concept has garnered significant interest in the realm of research [22]. It has three electrodes: drain (D), source (S), and gate (G) [23]. Among them, a conductive channel composed of semiconductors is formed between the drain and the source, and the carriers in the channel can be controlled by adjusting the electric field of the gate, thus controlling the current between the drain and the source. Due to the advantages, including higher sensitivity and high integration real-time detection, FET has been used in many fields to detect various types of analytes such as proteins, gas, small molecules, etc. However, the design of the gas-sensing interface disturbs the development of the FET enzyme electrochemical gas sensor. To the best of our knowledge, prior research into the electrochemical gas sensor based on the FET enzyme remains limited.

**Figure 3:**
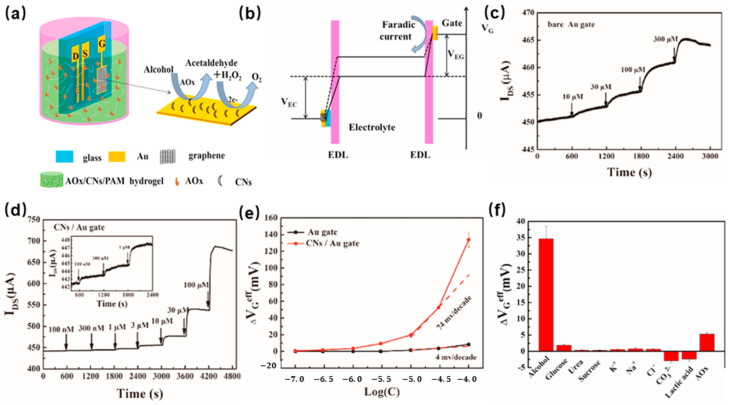
(**a**) Schematic of the solution-gated graphene transistors (SGGT) modified with AOx/CNs in a polyacrylamide hydrogel-based system; (**b**) The change in potential between the gate and channel before (solid line) and after (dotted line) the addition of alcohol in PBS solution; (**c**) the detection of increasing H_2_O_2_ concentration in PBS solution by a bare Au gate; (**d**) the detection of increasing concentration of H_2_O_2_ in PBS solution by CN-modified Au gate; (**e**) Change in effective gate voltage of the SGGT with unmodified Au gate and CN-modified Au gate vs. the logarithm of H_2_O_2_ concentration; (**f**) The selectivity of the SGGT device with the AOx/CN-modified gate [25].

Vianello et al. [24] designed an ion-sensitive field-effect transistor (ISFET) in conjunction with an aldehyde dehydrogenase specific for the detection of formaldehyde. The silicon nitride is applied for the gate dielectric of silicon-integrated ISFET. The formaldehyde dehydrogenase enzyme is immobilized on the surface of silicon nitride through covalent binding. In the sensing process, the nicotinamide adenine dinucleotide (NAD) was applied as a cofactor, catalyzing the oxidation of the formaldehyde molecule. The detection limit of this sensor is about 0.1 ppm. Zhao et al. [25] developed a breath alcohol biosensor based on a hydrogel-gated graphed field-effect transistor. The carbon nanosheets (CNs) were synthesized through fast pyrolysis of chlorella. Then, alcohol oxidase (AOx) was introduced into the substrate to detect alcohol in real time. The structure of the hydrogel-gated graphed field-effect transistor is shown in Figure 3a. There are three electrodes, namely gate, source, and drain, which contact the hydrogel. Due to the high carrier mobility feature of graphene enabling a significant field-effect amplification of the sensor, that was applied to construct the channel of the source and drain electrodes, the carbon nanosheets were employed to modify the gate electrode. The alcohol-sensing mechanism of the sensor depends on the oxidation reaction of alcohol by AOx and the electrocatalytic oxidation reaction of the generated H_2_O_2_. In detail, the enzymatically produced H_2_O_2_ will be oxidized on the surface of the Au gate electrode under a bias voltage. In this reaction, the electrons will be transferred to the gate electrode, and the Faradaic current will be generated by the redox reaction of H_2_O_2_ on the gate surface, which will cause the change of effective gate voltage, as shown in Figure 3b. The channel current response to H_2_O_2_ for the bare Au gate and CN-modified Au gate, as shown in Figure 3c,d. The CN-modified Au gate field-effect transistor alcohol oxidase electrochemical gas sensor exhibits a better sensitivity and lower limit of detection to 100 nM, 100 times lower than that of the device with a bare Au gate. As shown in Figure 3e, the effective gate voltage change vs. H_2_O_2_ concentration in logarithm. The CN-modified gate SGGT shows a response of 74 mV per decade, which is higher than that of the bare Au gate SGGT (4 mV per decade). Meanwhile, the CN-modified Au gate exhibits a dramatic enhancement in the current response. In addition, to verify the sensor’s specificity, interfering components were used in the detection of alcohol. As Figure 3f shows, the AOx/CN-modified gate sensor demonstrates excellent selectivity to alcohol. This sensor has been successfully applied to breath tests after alcohol drinking.

## 3. The Immobilization Method of Enzyme

The sensing interface plays a critical role in the electron transfer for enzyme catalysts. The process of enzyme sensing can be divided into two parts: gas molecular recognition, which correlates with the enzymes catalyzing gaseous chemical reactions; and signal transduction, which is related to the mechanism of enzymatic reaction. In consideration of electron transfer between an electrode and enzyme biocatalyst, it is important to rationally design an electrode surface to optimize the interaction between the enzyme and the electrode surface. Since enzymes that are used in gas biosensors are not in their natural surroundings, they tend to be less stable due to the changes in environmental conditions. If the bare enzyme (the enzyme that has not been immobilized in the substances) is modified on the electrode surface, the load amount of the enzyme becomes smaller, resulting in reduced enzyme activity. Furthermore, the electron transfer efficiency between the enzyme and the electrode will decrease. In the condition of enzyme gas sensor manufacturing, immobilization of the enzyme can enable close contact between the enzyme and the electrode surface while preserving the catalytic competence and avoiding the leakage of the enzyme into the sample. Meanwhile, in the process of enzyme immobilization, the introduction of substances should be avoided to block the specific sites of gas adsorption.

**Figure 4:**
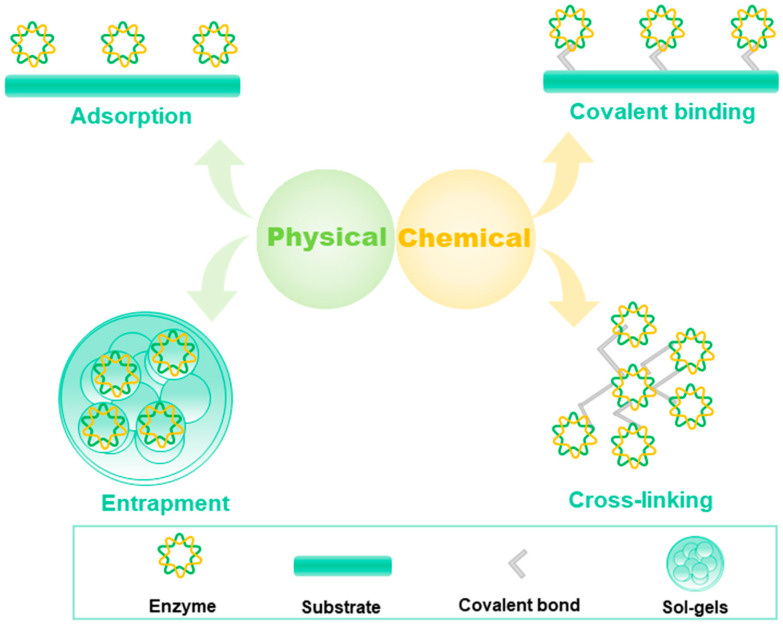
Physical and chemical methods for immobilization of enzymes.

The most common method currently used to address this is by immobilizing the enzyme onto the electrode surface. One approach to solving this problem is immobilizing enzymes in polymers. For example, polystyrene sulfonate can be dropped onto the electrode surface to immobilize oxidases [26], polyethyleneimine was applied to immobilize enzymes on agarose gels [27], bilirubin oxidase was introduced into a Nafion and crosslinked with glutaraldehyde to form a stable electrochemical interface [28]. The immobilization of enzymes can be classified as physical and chemical methods [29]. Adsorption [30], entrapment, and encapsulation are physical immobilization methods [31], whereas covalent bonding [32], crosslinking, and electrostatic attraction are classified as chemical immobilization methods (Figure 4), and the immobilization methods for enzyme-based electrochemical gas sensors were described in Table 1. In this section, we discuss the principles and latest developments of various immobilization methods.

### 3.1. Physical Adsorption 

Physical adsorption is based on van der Waals attractive forces between enzymes and electrodes, which is one of the first used and the simplest methods for enzyme immobilization [38]. It is normally sufficient for short-term studies but makes the enzyme electrode easily polluted. Jiang et al. [39] prepared an amperometric ethanol biosensor by integrating alcohol dehydrogenase with mediator meldola’s blue (MB). Based on the strong π–π stacking interaction between the aromatic group of MB and the graphene, MB was adsorbed on the surface of carbon nanotubes, and the loading, electron transfer kinetics of MB, and stability were improved. By adsorbing MB onto the carbon nanotube surface, the loading, electron transfer kinetics of MB, and stability were enhanced. The alcohol dehydrogenase-based sensor demonstrated a lower detection limit of 19.1 ±  0.58 μM and excellent selectivity. Unfortunately, the activity of the dehydrogenase sensor was only one week. In a separate study, Kundu et al [40]. developed an electrochemical formaldehyde dehydrogenase (FDH) enzyme sensor based on a screen-printed electrode for formaldehyde detection in corn. The formaldehyde dehydrogenase enzyme was immobilized on the α-Fe_2_O_3_/ITO electrode surface through physical adsorption. The fabrication process of the screen-printed electrode-based enzyme sensor involved preparing α-Fe_2_O_3_/ITO through electrophoretic deposition, followed by the addition of the enzyme onto the electrode surface and overnight incubation. Additionally, a cofactor (NAD+) was used in conjunction with FDH to enhance stability during the enzyme-catalyzed reaction. Subsequently, the enzyme electrode was treated with bovine serum albumin solution (BSA) to block nontargeted sites on the electrode surface. The enzyme sensor exhibited high sensitivity and low detection limits (0.03 mg/L). This immobilization method is simple and does not require chemical reactions, but the enzyme is easily inactivated.

### 3.2. Entrapment in Sol-Gels

Entrapment in sol–gels is as mild as adsorption, which is one of the major approaches for the immobilization of enzymes. This method involves caging the enzyme within a polymeric network through the formation of covalent or noncovalent bonds, which allow the passage of substrate and products but retain the enzyme. However, the two major issues obstruct the development of entrapment-based electrochemical enzyme sensors. One is the leaking of enzymes, and the other is the sluggish substrate–enzyme active site mass transfer [41].

To address these challenges, Adhikari et al. [42] proposed a new facile enzyme entrapment, a special cationic polymer poly(2-(dimethylamino)ethyl methacrylate) (MADQUAT) on single-wall carbon nanotube and reduced the graphene oxide (SWCNT–rGO) thin film to form an entrapment platform. Subsequently, alcohol dehydrogenase (ADH) is immobilized into the entrapment platform by the strong electrostatic affinity for the detection of ethanol. The entrapped alcohol dehydrogenase enzyme exhibits a high ability to transfer electrons and significantly enhances the enzyme catalytic activity. The developed ethanol sensor exhibits high sensitivity (26.27 μA mM^−1^ cm^−2^), and a low limit of detection (0.16 μM). Istrate et al. [10] designed an alcohol sensor by entrapping alcohol dehydrogenase into sol–gel matrix that was immobilized on the surface of the screen-printed electrode (SPE) modified with poly(allylamine hydrochloride). Das et al. [43] developed a direct electrochemistry enzyme sensor for the detection of alcohol. An alcohol oxidase (AOx) was immobilized on a multiwalled carbon nanotubes-Nafion (MWCNT-Nf) matrix and encapsulated with polyethylenimine (PEI) on the gold electrode (AuE). The surface morphology of bare Au shows a homogenous surface. MWCNTs with porous morphology uniform distribution on the electrode surface. When AOx was added to this film, the porosity disappeared, indicating the AOx was immobilized on the MWCNT-Nf film. Then, the alcohol oxidase (AOx) was immobilized on multiwalled carbon nanotubes-Nafion and encapsulated with polyethyleneimine (PEI) on the surface of the electrode. The electron was transferred directly between the AOx and the electrode. Meanwhile, the entrapped AOx presented good bioactivity and electrocatalytic activity. The AOx enzyme sensor has a rapid response time of 55 s, a low detection limit of 5 μM, and exhibits potential applications for detecting alcohol in real samples. Hiroyuki et al. [36] designed a choline oxidase-based choline vapor sensor, which was fabricated by entrapping the choline oxidase in sol–gels on a Clark-type dissolved oxygen electrode, this sensor shows excellent choline gas-sensing performances. These findings provide a new paradigm for the design of enzyme electrodes. This method can protect the enzyme from the external environment but may reduce the reaction rate of the enzyme.

### 3.3. Covalent Coupling

**Figure 5:**
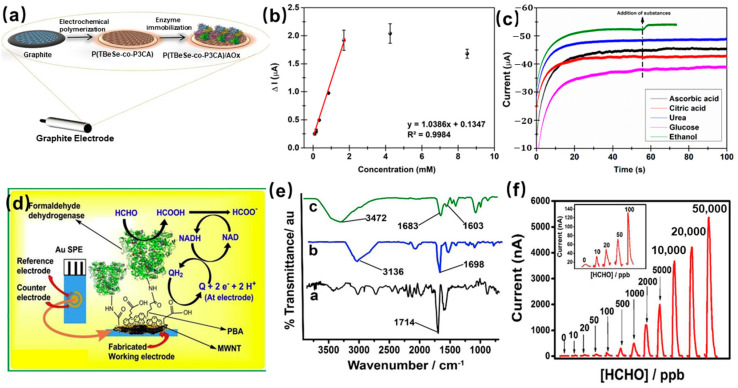
(**a**) Schematic of sensing device containing graphite electrode, a network of polymer and AOx; (**b**) Amperometric response of the alcohol sensors to increasing ethanol concentrations; (**c**) Effect of interfering substances on biosensor performance [44]; (**d**) the immobilization of the formaldehyde dehydrogenase and the mechanism for catalyzing formaldehyde; (**e**) The FTIR spectra of formaldehyde dehydrogenase immobilized on pyrenyl carbon nanostructures; (**f**) Amperometric responses for various concentration of formaldehyde [37].

Covalent coupling is one of the most promising methods in the process of enzyme immobilization for the design and construction of enzyme-based electrochemical sensors. Covalent coupling provides a potential way to preserve enzyme activity for a long period. This method mainly depends on the formation of a covalent bond between the enzyme and the support material. Soylemez et al. [44] designed a newly synthesized copolymer with enhanced enzyme-sensing properties as a novel sensor for the detection of ethanol. The conjugated copolymer (TBeSe-co-P3CA) was prepared on the surface of the electrode by electrochemical polymerization. After the alcohol oxidase (AOx) was immobilized through the covalent linkage between the enzyme’s amino group and the carboxyl group derived from P3CA, the schematic of the sensing device and the preparation process is shown in Figure 5a. The prepared sensor exhibits excellent ethanol sensing performance, as Figure 5b shows; the amperometric response increases and then reaches the steady-state value, the limit of detection is 0.37 mM. Meanwhile, the sensor specificity was measured against interfering compounds, including ascorbic acid, citric acid, urea, glucose, and ethanol. It can be seen from Figure 5c that the sensor exhibits a distinct change of current to ethanol, indicating excellent specificity. Soylemez et al. [45] constructed the enzyme electrode by immobilizing the AOx onto the carbon nanotubes modified conducting polymer with the help of EDC (1-ethyl-3-(3-dimethylaminopropyl)carbodiimide)/NHS (N-hydroxysuccinimide) crosslinking, the preparation of biosensor. EDC and NHS were used to activate the free carboxylic acid groups of the conducting nanotube, and the amide bond formed between enzyme molecules and carbon nanotubes for the covalent attachment of enzymes. The XPS result confirmed the formation of the covalent bond. This alcohol oxidase-based sensor has been successfully used for the detection of alcohol, with a lower limit of detection is 0.17 μM.

The other example of an enzyme immobilized by covalent coupling is as follows: the first pyrenyl carbon nanostructure-based enzyme gas sensor for urine formaldehyde quantitation was fabricated by Gayan et al. [37]. The NAD+-dependent formaldehyde dehydrogenase (FDH) was immobilized by covalent coupling on the surface of the carboxylated multiwalled carbon nanotubes stacked with π–π 1-pyrenebutyric acid units, the fabrication steps, and the mechanism for catalyzing formaldehyde as Figure 5d shows. EDC and NHS were used to activate the free carboxylic acid groups of the conducting nanotube, and the amide bond formed between enzyme molecules and carboxylated multi-walled carbon nanotubes for the covalent attachment of enzymes. The FTIR spectra results confirmed the formation of the covalent bond (Figure 5e). The appearance of a new board peak at 3472 cm^−1^, indicates the covalent immobilization via the formation of an amide bond between the carboxylated multiwalled carbon nanotubes and formaldehyde dehydrogenase. This sensor shows an excellent formaldehyde-sensing performance, as Figure 5f shows. The biosensor provides an ideal immobilization matrix for the formaldehyde dehydrogenase and has been successfully used for the detection of formaldehyde samples in urine with satisfactory results. However, the fabricated formaldehyde dehydrogenase-based sensor, with a short lifetime of 30 h, is under continuous testing. In conclusion, this method can immobilize the enzyme stably but may affect the activity and stability of the enzyme.

### 3.4. Crosslinking Method

Crosslinking is a method of immobilizing enzymes on a carrier surface. In this method, chemical crosslinking agents are used to bind the enzyme to the functional groups on the carrier surface, forming a chemical bond to immobilize the enzyme. Glutaraldehyde is one of the commonly used crosslinking agents. Enzyme–chemical crosslinking has advantages such as high efficiency of enzyme immobilization, strong binding force between the enzyme and the carrier, and good stability. Additionally, this method can control the density and position of the immobilized enzyme and adjust the chemical properties of the carrier surface to further improve the catalytic efficiency and stability of the enzyme.

**Figure 6:**
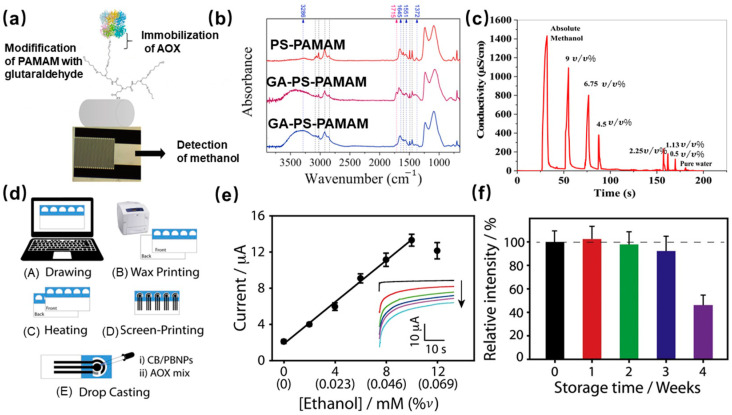
(**a**) The schematic of preparation alcohol oxidase-based methanol sensor; (**b**) FTIR spectra of PS-PAMAM, GA-PS-PAMAM, and AOX/PS-PAMAM ESNFs samples; (**c**) the response of sensor to a series concentration of gas-phase methanol [35]; (**d**) The schematic diagram of the process for preparing paper-based ethanol biosensor; (**e**) The calibration plot of ethanol concentration vs. current (The inset shows the chronoamperometric records with the concentration increase from 0–12 mM); (**f**) the evaluation of shelf-life at 4 °C of the ethanol sensor, fresh biosensor (black bar), 1 week (red bar), 2 weeks (green bar), 3 weeks (blue bar) and 4 weeks (violet bar) [19].

Razmshoar et al. [35] created a conductometric enzymatic methanol sensor by grafting alcohol oxidase (AOx) onto electrospun polystyrene-poly(amidoamine) dendritic polymer nanofibers. To form a strong link between AOx and the surface of nanofibers, glutaraldehyde coupling was used to form a bond between the amine groups of the AOx enzyme and the nanofiber dendritic polymer groups (Figure 6a). The FTIR spectra, which were used to evaluate the chemical structure, showed a peak at 1674 cm^−1^. This peak is assigned to the C=N group and confirms the covalent link between the polystyrene-poly(amidoamine) and the glutaraldehyde. Notably, this peak is due to the binding between the AOx and glutaraldehyde, as depicted in Figure 6b. This sensor was applied for the detection of gaseous methanol concentrations above different methanol/water solutions (Figure 6c). The sensor showed rapid response/recovery performance: the response time was from 13 s to 35 s, the recovery time from 6 s to 10 s was from a lower concentration of methanol to its higher concentrations, and the detection limit was 100 ppm. Niculescu et al. [46] have developed an ethanol biosensor based on dehydrogenases, Poly(vinyl imidazole) complexed with Os(4,40-dimethyl bipyridine)_2_Cl employed as the electrochemical mediator, and poly(ethylene glycol)-diglycidyl ether as the crosslinking agent. The developed enzyme sensor has been successfully employed for the detection of ethanol in wines. Cinti et al. [19] reported a paper-based electrochemical ethanol biosensor. The electrode was modified by carbon black (CB) and Prussian blue nanoparticles (PBNPs), and then the AOx enzyme was immobilized via crosslinking, the fabrication route is shown in Figure 6d. The ethanol-sensing performance as Figure 6e shows, the chronoamperometric records of ethanol concentration up to 10 mM in 100 μL of 0.05 M phosphate-buffered solution containing 0.1 M KCl at pH 7.4, displaying a very quick and sensitive response after just 40 s. Meanwhile, the shelf-life of the biosensor was evaluated, as Figure 6f shows no significant decrease in initial activity after storage of 3 weeks, but the response decreased by 50% after the fourth week. This method can improve the stability and reaction rate of the enzyme but may affect the activity of the enzyme.

## 4. Electrodes Materials Selection and Modification

The transducer in the electrochemical biosensor is the electrode. The electrochemical enzyme-based gas sensor converts chemical signals into electrical signals. The electrode has a high sensitivity to gas and is easy to modify. Recently, advances in nanostructure and material have been applied to enhance electrochemical performance. The electrode materials selection and modification are to achieve efficient electron transfer between the electrode and the gas to be detected and to reduce the interference between the sensor and other environmental factors. The materials and modifications of the electrodes are discussed as follows:

### 4.1. Selection and Design of Surface-Modified Materials

The immobilization of the enzymes on the electrode surface is considered one of the critical steps that dictates the effectiveness of the enzyme electrodes. The special functional chemical materials are modified on the electrode surface, which can enhance the interaction between the enzyme and the electrode, and the sensitivity can be improved. Recently, the application of sol–gel [47], polymers [48], nanomaterials [49], and composite materials [50] for the construction of efficient enzyme electrodes has become a popular research area.

Surface-modified materials for the electrode are a crucial element of the amperometric enzyme gas sensor. The selection of suitable material combinations for the construction of enzyme-modified electrodes dictates the efficiency of the enzyme electrodes in terms of electron transfer kinetics, gas diffusion, stability, and reproducibility. Conducting polymers are extensively utilized due to their dual advantages of binding the active enzyme and facilitating suitable electron transport in enzyme electrodes. Another application of polymers is functionalized polymer, which is employed in the assembly of enzyme electrodes. In this process, a polymer functionalized with an amino group is electrodeposited onto the electrode surface, followed by the subsequent coupling of biomolecules through the activation of the protein’s carboxylic group using carbodiimide crosslinking [51]. Sol–gel is a polymer network system that utilizes water as a dispersion medium. It has strong water absorption, water retention, and mechanical properties, along with exceptional biocompatibility and ion transfer capabilities. Sol–gel serves as a versatile medium for encapsulating bioactive molecules like enzymes and coenzymes, preserving their activity for various sensor applications [52]. This is achieved through physical encapsulation and immobilization of the enzyme within the polymer mesh matrix. According to reports, the appropriate incorporation of polyethylene glycol, polyvinyl alcohol, and albumin in sol-gel has been shown to significantly enhance the stability of the enzyme in the substrate [53]. At the same time, nanomaterials offer a range of nanoscale materials for enzyme immobilization. In contrast to traditional large-size materials, nanomaterials also possess the benefits of a large specific surface area, straightforward surface modification, and a size similar to enzyme molecules. Nanomaterials, as a novel type of enzyme immobilization carrier, have garnered significant attention in the realm of biocatalytic technology. Immobilized enzymes not only exhibit a high enzyme loading capacity but also demonstrate excellent enzyme stability. For example, the use of Au nanoparticles as a highly significant enzyme carrier has generated increasing research interest. Gold nanoparticles, as immobilized enzyme carriers, have the following advantages: (1) a straightforward preparation method and easy to regenerate; (2) the excellent biocompatibility of Au is maintained, effectively preventing enzyme inactivation; and (3) direct binding of the amino group in the enzyme molecule and the sulfhydryl group in cysteine to the surface of gold nanoparticles for enzyme immobilization.

Sundari et al. [54] investigate the use of nanogold doping in the p-HEMA membrane for the detection of formaldehyde. Nanogold particles act as conduction centers to facilitate electron transfer in the formaldehyde reaction at the electrode surface. Carbon-based nanomaterials, such as graphene and carbon nanotubes have been widely used in the field of biosensors due to their advantages of high conductivity, good biocompatibility, and chemical inertia. Li et al. [48] designed an amperometric alcohol dehydrogenase biosensor for ethanol based on electroreduced graphene oxide–polythionine nanocomposite film. The electroreduced graphene oxide and polythionine film shows an efficient electrocatalytic characteristic toward the oxidation of β-nicotinamide adenine dinucleotide. Meanwhile, the sensor exhibits great potential for ethanol detection.

### 4.2. Electrode Materials Property and Optimization

The enzyme is the kingpin of enzyme electrodes. The enzyme biocatalyst is mostly chosen to construct electrodes for gas molecules, which exhibit high specificity and are essential for gas molecules. Due to the unique electrical and catalytic properties and good biocompatibility, nanomaterials have been applied for electrode modification materials, which have high activity and selectivity. Nanomaterials have been employed to modify the electrode, which exhibits a larger specific surface area and excellent adsorption performance. Thus, the current response is improved, and the limit of detection is decreased. At present, carbon nanotubes [55], graphene [56], MXene [57] mesoporous silica materials, etc. [58] are widely used for electrode materials.

For example, due to low cost and simple preparation procedure, polymer membranes are extensively used as the supporting materials in the development of biosensors, which can be used as trappers for various substrates [59]. Nurlely et al. [60] described a membrane-based potentiometric biosensor that has been fabricated from poly(n-butyl acrylate-co-N-acryloxysuccinimide (pnBA-NAS) as an enzyme-supporting matrix on the Ag/AgCl screen-printed electrode for rapid and facile determination of formaldehyde. pnBA-NAS applied for the H^+^ ion transfer at the electrode-electrolyte interface. The H^+^ ion transducer membrane detects pH change from the enzymatic reaction and gives an EMF signal, which can quantify the concentration of formaldehyde.

In the selection of the electrode materials, new requirements are put forward for nanomaterials. The nanomaterial with a bigger surface area provides more catalytic active sites. Proper orientation of enzymes that were modified on the electrode must be designed, which can reduce the active site of the embedded state and improve the effectiveness of electron transferring. Meanwhile, the nanomaterials must have better biocompatibility, which is conducive to the fixation of enzymes and provides enough microenvironment for biochemical reactions. Moreover, the instability of the enzyme electrodes restricts the operation in nonaqueous environments and at high temperatures. Recent advances in interface engineering provide an opportunity to design the enzyme electrode with novel properties. In the construction of enzyme electrodes, materials that are close to the enzymes should be considered to improve the enzyme activity. Meanwhile, the operating conditions, mechanical and chemical properties, biocompatibility, and low cost for mass production are some of the important features considered for the construction of enzyme electrodes. Additionally, an ideal electrode material must be characterized by good conductivity to ensure rapid electron transfer.

## 5. The Charge Transfer Mechanism of the Enzyme Electrode

Nowadays, there are several challenges in the development of enzyme-based electrochemical gas sensors. One of the biggest challenges is the target gas coupling to the surface of the electrode. The electron transfer restricts the transduction efficiency of the sensor. The enzyme is a biological macromolecule, its electroactive group or center is embedded in the polypeptide chain, and the electron transfer rate is limited. The development of electrochemical electrodes can help overcome these challenges.

### 5.1. Electrodes with Mediated Electron Transfer

**Figure 7:**
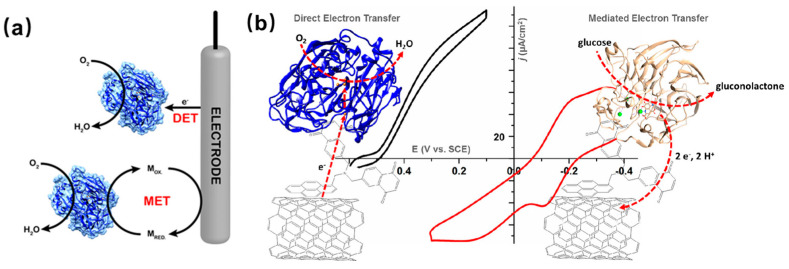
(**a**) Reduction of O_2_ to H_2_O by bilirubin oxidase via direct electron transfer (DET) or mediated electron transfer (MET); (**b**) Electrodes modified with naphthoquinone functionalized pyrene/carbon nanotubes can promote direct electron transfer to laccase and bilirubin oxidase, as well as PQQ-dependent glucose dehydrogenase-mediated electron transfer-dependent glucose dehydrogenase-mediated electron transfer [32].

Oxidoreductases are widely utilized for catalyzing the transfer of electrons, and various enzymes have been explored for gas detection, as Table 2 shows. This process can occur through either direct electron transfer (DET) or mediated electron transfer (MET), as depicted in Figure 7a,b. The type of transfer that can occur is associated with the type of enzyme. In the process of mediated electron transfer, which is mainly based on the utilization of low-molecular redox mediators. The enzyme catalyzes the oxidation or reduction of the mediator, and the mediator undergoes redox transformation or regeneration on the electrode’s surface. Mediator-assisted electron transfer is defined by two primary characteristics. First, the mediator must function as a secondary substrate for the enzymatic reaction. Second, the electrochemical conversion of the mediator on the electrode must be reversible. In this process, both the analyte and the mediator undergo enzymatic transformations during the catalytic process. The mediator is then regenerated at the electrode surface without requiring overvoltage. The electrode process for mediator regeneration is noncatalytic, allowing the enzymatic transformation and electrode reaction to be treated as separate reactions within the overall coupled process. The choice of mediator is a critical factor in catalyzing gas reactions. An effective mediator should remain stable under working conditions and not engage in side reactions during electron transfer. An ideal mediator can lower the potential of chemical reactions and enhance the speed of electron transfer [61]. Consequently, the efficiency of gas reactions catalyzed by enzymes is improved. Reducing the potential through the medium can prevent the passivation of the enzyme electrode. The mediator must be soluble in both its oxidized and reduced forms, enabling rapid diffusion between the enzyme’s active site and the electrode surface. Generally, low molecular weight mediators such as Ferrocene [61], Prussian blue [62], ferricyanide [63], 1,2-naphthoquinone-4-sulfonic acid [64], and others are commonly used in gas detection, primarily influencing the diffusion of gas onto the surface of the enzyme electrode.

Prada et al. [71] reported a graphite–Teflon composite enzyme amperometric biosensor for monitoring alcohols. Alcohol oxidase and horseradish peroxidase, as well as the mediator ferrocene, are incorporated into the electrode matrix. In the process of detection, the amperometric signal corresponded to the electrochemical reduction of ferricinium. Li et al. [72] have described a facile method to chemically reduce graphene functionalized with polythionine to mediate electron transfer in biosensors, especially for H_2_O_2_ and NADH detection, which is employed for the detection of ethanol. The mediator can be covalently or noncovalently bound to the backbone of the enzyme. In the previous work, the ferrocene/ferrocenium redox couple dissolved in solution to transport electrons from glucose oxidase [73]; in this process, the flavin adenine dinucleotide (FAD) cofactor of glucose oxidase is reduced by the enzymatic oxidation of glucose. At the same time, ferrocene is reduced at the enzyme’s active site, and the reduced FADH_2_ cofactor returns to its oxidized state, promoting further oxidation of glucose.

In the measurement of acetone concentration in human breath, an acetone-sensitive enzyme system was utilized [12]. The acetone is oxidated to hydrogen peroxide (H_2_O_2_) via secondary alcohol dehydrogenase (s-ADH) and an NADH cofactor. The enzyme mixture contains the three enzymes: s-ADH, lactate dehydrogenase (LDH), and pyruvate oxidase (PO). In the acetone sensing process, NAD^+^ and NADH as mediators, acetone is first reduced to 2-propanol by the s-ADH, meanwhile, NADH converts to NAD^+^. Then, in the presence of LDH, the NAD^+^ converts lactate to pyruvate. Finally, in the presence of PO, pyruvate is converted to acetyl phosphate, H_2_O_2,_ and CO_2_ forms. The H_2_O_2_ can be detected by amperometry, and the measured current is proportional to the concentration of breath acetone. In an alcohol dehydrogenase-based electrochemical enzyme gas sensor, ethanol is oxidized to acetaldehyde by a coenzyme, nicotinamide adenine dinucleotide (NAD^+^), which is necessary to accept electrons from ethanol with the catalyst of ADH [39]. The NAD^+^ is reduced to NADH. Due to the sluggish charge transfer kinetic of direct electrochemical oxidation at convention electrodes and the fouling of electrodes. An electron mediator was introduced to accelerate the electron transfer rate between NADH and the conducting substrate. 8-dimethylamino-2, 3-benzophenoxazine (meldola’s blue, abbreviated to MB) was selected as an effective electron relay for NADH. The electron transfer kinetics and the stability are enhanced by the loading of MB. The ethanol sensor exhibits excellent sensitivity and selectivity performance. Rosmarinic acid, as a new redox mediator is employed for the modification of carbon electrodes for electrochemical determination of ethanol. Alcohol dehydrogenase (ADH)-based ethanol senor catalyzes the oxidation of ethanol in the presence of the cofactor adenine dinucleotide (NAD^+^) into β-nicotinamide adenine dinucleotide (NADH). The concentration of ethanol is determined by its electrooxidation. Unfortunately, the electrooxidation of NADH on bare electrodes requires high potentials, which results in contamination of the electrode surface. Modifying the electrode with mediators can effectually reduce overpotential. Rosmarinic acid as an antioxidant, has been explored to modify electrodes as an ethanol sensor. The presence of rosmarinic acid in the enzyme sensor caused the ethanol detection analysis at relatively low potentials, the operational stability was enhanced, and the risk of interferences was decreased [74].

Despite mediators having many advantages, some other factors restrict the development of enzyme-based electrochemical gas sensors, such as the consumption of enzymes during the reaction, the leaching of the mediator, and the toxicity of the mediators.

### 5.2. Electrodes with Direct Electron Transfer

Nowadays, biosensors without a mediator in the process of electron transfer have been explored. In general, there are either redox-active cofactors or metal centers contained in a redox-active enzyme, which allow direct communication between the enzyme and the electrode (DET). However, achieving direct electron transfer between the enzyme and the electrode surface is normally challenging. For the reaction of DET, a short distance (<20 Å) [32] is necessary. An essential element in creating an effective electrochemical enzyme gas sensor with high sensitivities, high specificity, and fast response is the establishment of a fast electron transfer from the enzyme to the electrode. In the process of designing and constructing an enzyme-based electrochemical gas sensor, it is necessary to shorten the distance between the surface of the electrode and the immobilized enzyme. The development of chemical engineering techniques and the modification of genetic techniques provide an opportunity for the design of the highly efficient and sensitive direct electron transfer electrochemical enzyme-based gas sensor [13,72]. Kumar et al. [75] have designed an enzyme electrode for direct electron transfer, which is applied for the detection of ethanol. Alcohol oxidase was immobilized through the layer-by-layer method, carbonylated graphene, and alcohol oxidase on poly diallyl dimethyl ammonium chloride applied modified graphite electrode. DET of alcohol oxidase was observed. The enzyme electrode exhibited electrocatalytic activity in the reduction of oxygen, with the reduction current decreasing linearly as the ethanol concentration increased, indicating its potential use in mediator-free ethanol biosensors.

## 6. Application and Prospect of Enzyme-Based Electrochemical Gas Sensors

In summary, the design and construction of enzyme-based electrochemical gas sensors is a comprehensive work, involving surface modification, enzyme immobilization, mediator selection, and electrode material optimization. The reasonable design of the enzyme electrode is the critical factor for improving the performance and sensitivity of the sensor. The proper design of the enzyme electrode is beneficial to the transfer of charge, the stabilization of the interface of the enzyme electrode, and the lifetime of the enzyme. Although a great deal of progress has already been made in the interface between the enzyme and the electrode, there remain several challenges and obstacles to the electron transfer of enzyme electrodes, and the activity of enzymes.

From the perspective of materials, to combine structural–functional materials and sensing enzyme materials well, they have excellent combination characteristics, which are beneficial for the construction of an all-solid-state enzyme-based chemical gas sensor. In the future, it is promising to develop the enzyme gas sensor where direct electron transfer between the enzyme and the electrode is established to generate the response, which exhibits the major advantages interference-free (due to the operating potential window closer to the redox potential of the enzyme), and less modified materials or reagent. Meanwhile, more comprehensive, and in-depth characterization, performance testing, and theoretical calculation studies are needed to explain the specific sensing response behavior. Additionally, the development of nanozymes has brought new opportunities for the development of enzyme-based electrochemical gas sensors. Nanozymes are considered one of the new functional materials, which have both physical and chemical properties and optical, electrical, and magnetic properties of nanomaterials, as well as unique enzyme-like catalytic activity. Gas sensing based on nanoenzymes will be the next research focus.

## Figures and Tables

**Table 1:** The immobilization methods for enzyme-based electrochemical gas sensors.

Gases	Enzyme	Immobilization Methods	Ref.
Ethanol	Alcohol oxidase	Entrapment	[25]
Carbon dioxide	Carbonic anhydrase	Entrapment	[33]
Lactic acid	Lactate oxidase	Physical adsorption	[34]
Methanol	Alcohol oxidase	Crosslinking	[35]
Choline	Choline oxidase	Entrapment	[36]
Formaldehyde	Formaldehyde dehydrogenase	Covalent Coupling	[37]

**Table 2:** Various enzymes for gas detection.

Gases	Enzyme	LOD	Materials	Ref.
Acetone	Secondary alcohol dehydrogenase	0.25 ppm	-	[12]
Ethanol	Alcohol oxidase	46 ppb	Hydrogel	[25]
Phenol	Polyphenol oxidase	29 ppb	Hydrogel	[65]
Hydrogen peroxide	Catalase	-	Polyvinyl alcohol	[66]
Ethanol	Alcohol oxidase	55 ppb	-	[67]
Methyl mercaptan	Monoamine oxidase	-	-	[68]
Formaldehyde	Alcohol oxidase	0.15 ppm	Poly(allylamine)	[69]
Toluene	Butyrylcholinesterase	-	Polyvinyl alcohol	[70]

## Data Availability

Not applicable.
